# Assessment of the current Canadian rhinology workforce

**DOI:** 10.1186/s40463-015-0070-x

**Published:** 2015-05-09

**Authors:** Kristine A Smith, Doron D Sommer, Sean Grondin, Brian Rotenberg, Marc A Tewfik, Shaun Kilty, Erin Wright, Arif Janjua, John Lee, Chris Diamond, Luke Rudmik

**Affiliations:** Division of Otolaryngology, Head and Neck Surgery, Department of Surgery; University of Calgary, Calgary, Alberta Canada; Division of Otolaryngology, Head and Neck Surgery, Department of Surgery, McMaster University, Hamilton, Ontario Canada; Division of Thoracic Surgery, Department of Surgery, University of Calgary, Calgary, Alberta Canada; Department of Otolaryngology, Head and Neck Surgery, University of Western Ontario, London, Ontario Canada; Department of Otolaryngology, Head and Neck Surgery; McGill University, Jewish General Hospital, Montreal, Quebec Canada; Department of Otolaryngology, Head and Neck Surgery, University of Ottawa, Ottawa, Ontario Canada; Division of Otolaryngology-Head & Neck Surgery, University of Alberta, Edmonton, Alberta Canada; Division of Otolaryngology, Head and Neck Surgery, Department of Surgery, University of British Columbia, Vancouver, BC Canada; Department of Otolaryngology, Head and Neck Surgery, University of Toronto, Toronto, Ontario Canada

**Keywords:** Rhinology, Sinusitis, Rhinosinusitis, Workforce, Otolaryngology

## Abstract

**Background:**

The Canadian Rhinologic workforce and future needs are not well defined. The objective of this study was to define the current demographics and practice patterns of the Canadian Rhinologic workforce. Outcomes from this study can be used to perform rhinologic workforce needs assessments.

**Methods:**

A national survey was administered to all Canadian otolaryngologists who were identified to have a clinical practice composed of >50% rhinology.

**Results:**

42 surgeons participated in the survey (65% response rate). The mean age was 46 (SD 10.1) years and the average age of planned retirement was 66 (SD 4.0). Eighty three percent of respondents had completed a rhinology fellowship and 17% practiced exclusively rhinology. Thirty three percent hold advanced degrees. Forty two percent of surgeons felt their access to operative time was insufficient. Six percent of surgeons reported not having access to image guided surgery. Fourteen percent felt that there were too many practicing rhinologists in Canada while 17% believed there were too few practicing rhinologists. Seventeen percent have advised their residents to pursue other fields due to a perceived lack of future jobs. Overall, 66% of respondents were satisfied with their income, and 83% were satisfied with their careers.

**Conclusions:**

This study has demonstrated that there is a perceived mismatch between the current supply of Rhinology labor and the capacity to treat patients in a timely manner. Outcomes from this study will begin to improve Rhinologic workforce planning in Canada and reduce the gap between patient demand and access to high quality care.

## Introduction

Workforce planning is a relatively new approach to identifying the supply and demand of labor [1]. The goal of workforce planning is to identify talent surpluses and shortages, and project future needs to avoid similar issues. It also facilitates matching training to societal needs. In Canada, the lack of jobs in surgical subspecialties is well known. In 2013, the Royal College of Physicians and Surgeons of Canada reported the unemployment rate of recently graduated specialists was approximately 16% [2]. More specifically, of newly graduated otolaryngologists, almost 30% were unable to find permanent employment, despite a declining otolaryngologist to population ratio [2,3]. However, even with this apparent ‘surplus’ of surgeons, operative and consultation wait times continue to grow [4]. Deficiencies in the appropriate matching of workforce needs combined with reduced capacity to treat patients in a timely and accessible manner significantly reduces the quality of care and may adversely affect patients’ health [4-7]. Some of these issues would likely be resolved by improving the concordance between societal health needs and labor capacity.

All previous attempts to define the otolaryngology workforce have focused on the overall population of otolaryngologists, not individual subspecialties [3,8-11]. With an increasing number of residents pursing fellowship training, characterizing the subspecialist populations is paramount to create accurate workforce planning projections and improving efficient allocation of scarce health care resources [2,8]. Rhinology is a growing subspecialty within otolaryngology and this population has not been previously defined.

The purpose of this national survey was to describe the contemporary demographics, medical education, practice patterns, and surgeons’ attitudes towards workforce requirements and training of the current Canadian rhinologic workforce. Outcomes from this study will provide the necessary information required for physician workforce modeling and allow us to begin strategically planning for Canada’s future rhinologic needs.

## Methods

### Study design

This was an online survey-based study that invited participants between June and Aug 2014.

### Participant sample

Otolaryngologists with large volume rhinology practices (at least 50% rhinology) were selected for inclusion in the study. We chose a cut off of 50% rhinology since the practice patterns of these otolaryngologists would likely have the largest impact on the overall rhinology workforce. For this study we defined “rhinology” to include all endonasal surgery and excluded the practice of managing nasal conditions which require a rhinoplasty. In order to coordinate across all the provinces and territories in Canada, a representative rhinologist was selected from each province by the principal investigator (LR). Potential participants were identified through a review of the Canadian Society of Otolaryngology – Head and Neck Surgery (CSO) database by the provincial representatives. Email addresses were obtained from the CSO registry where available and otherwise were identified from the provincial representatives.

### Survey

The rhinology workforce survey was developed from the validated ‘Canadian thoracic surgery workforce survey’ using a modified Delphi technique by a panel of experts [12]. Questions from their survey were altered to reflect rhinology as the surgical specialty of interest, versus thoracic surgery. Any questions modified more than changing the surgical field of interest were reviewed using the Delphi technique until all authors agreed on the proposed question. The final survey consisted of 54 questions and was designed to collect data regarding demographics, education, current practice patterns, income, job satisfaction and future planning, and a basic workforce assessment. The survey was distributed using an online survey host, “Survey Monkey”, a web survey company located in the USA (http://www.surveymonkey.com/).

### Ethics, consent and permissions

Institutional ethics review board approval was obtained (ID: # REB14-0970). An email containing a link to the survey and an invitation to participate in the workforce survey was sent to all the otolaryngologists identified to have large volume rhinology practices (>50% rhinology). Participation was entirely voluntary. Two reminder emails were sent, the first one week after the initial invitation and the second two weeks after the initial invitation. Submission of the completed survey was considered informed consent to participate. No identifying data was collected and all survey responses were completely anonymous.

### Outcomes

The primary outcome was to define the demographics of the current rhinologist workforce in Canada. Secondary outcomes included: education, medical training, current practice patterns, scope of practice, income and job satisfaction with rhinology.

### Statistical analysis

Data was exported into Excel (Microsoft, 2010) for analysis. Data was correlated to survey results for accuracy and logic by one of the authors (KS). Descriptive statistics were calculated where appropriate.

## Results

A total of 65 otolaryngologists were identified as having large volume rhinology practices and were invited to participate in this study. Forty two (42) responded to the survey, representing a 65% response rate. Participants answered an average of 90% of questions, with participation decreasing towards the end of the survey.

### Demographics and education

Table 1 summarizes the demographic data of the respondents, including gender, age, practice location, country of birth and society memberships. The majority of respondents were male (91%), with an average age of 46 (SD = 10.1). Only 29% of respondents were over 50 years of age.

**Table 1:** **Demographics**

Gender	
Male	91%
Female	9%
Age (years) (SD)	
Average	46 (10.1)
<40	40%
40-50	31%
50-60	21%
>60	8%
Practice Location	
British Columbia	14%
Alberta	24%
Quebec	17%
Ontario	43%
Nova Scotia	2%
Country of Birth	
Canada	72%
USA	0%
Other*	28%
Memberships	
CSO	97%
ARS	52%
ERS	24%

*Egypt, China, Kenya, Ireland, England, Iran, Israel, Scotland and Austria.

*SD* - Standard Deviation.

*USA* – United States of America.

*ARS* – American Rhinologic Society.

*ERS* – European Rhinologic Society.

*CSO* – Canadian Society of Otolaryngology – Head and Neck Surgery.

The details of respondents’ medical training are summarized in Table 2. All surgeons had completed residency in Canada. 83% of respondents reported completing a rhinology fellowship. Forty six percent (46%) of fellowship trained rhinologists completed fellowship training in Canada, while 31% had completed fellowships in the United States (US). For rhinologists with a fellowship, the average year for beginning practice was 2005. For those who had not pursued a fellowship, the average year for starting their rhinology practice was 1991. Thirty three percent (33%) of surgeons had completed advanced graduate degrees (MSc, MPH, or PhD), with an additional 5% currently pursing post-graduate education.

**Table 2:** **Education**

Year of completion of (SD)	
Medical School	1995 (10.3)
Residency	2001 (9.9)
Fellowship Training	2005 (8.2)
Fellowship training	
Rhinology	83%
None	17%
Location of Fellowship Training	
Canada	46%
USA	31%
Other*	23%
Advanced Degrees	
Masters degree	33%
Masters degree (in progress)	5%

*Australia, Egypt, France, New Zealand, Belgium, Armenia.

*SD* – Standard Deviation.

*USA* – United States of America.

### Current practice patterns

The practice patterns of the respondents are outlined in Table 3. Surgeons reported dedicating over 75% of working hours to clinical and operative time, with the remaining quarter split between research, administrative duties and teaching. Approximately half (54%) were affiliated with a university, with the remainder in community practices. Seventy eight percent (78%) of respondents were involved in some form of research, with 32% involved in multiple types of research including educational, basic sciences, clinical outcomes, and health economics research. Eighty five percent (85%) reported at least one other surgeon performing rhinologic surgeries at their institutions.

**Table 3:** **Practice Patterns**

Language of Practice	
English	90%
French	20%
Cantonese	2%
Scope of Practice	
Exclusively Rhinology	17%
>75% Rhinology	30%
<75% Rhinology	53%
Distribution of work hours	
Clinical Work	76%
Research	8%
Administration	9%
Teaching	7%
Participation in Research	
Clinical	78%
Educational	32%
Basic Science	24%
Health Economics	5%
None	22%
Hospital Setting	
Community Hospital, no residents	22%
Community Hospital, residents	24%
University Hospital, residents	35%
University Hospital, residents and fellows	19%

Work hour requirements are outlined in Table 4. The majority of surgeons work 41-60 hours per week (61%), with 25% working over 60 hours per week. Forty four percent (44%) of respondents reported on-call requirements of 1 in 4 - 6 and an additional 44% reported call requirements of 1 in 7 -10. A majority, 66% of respondents, reported taking at least 3 to 5 weeks of vacation time and 98% of surgeons reported taking at least 3 to 7 days for continued medical education in the last year.

**Table 4:** **Work Hours and Vacation Time**

Work hours per week	
≤40	12%
41-50	27%
51-60	34%
61-70	12%
71-80	9%
81-90	2%
>90	2%
On call frequency	
1:1 to 1:3	2%
1:4 to 1:6	44%
1:7 to 1:10	44%
>1:10	10%
Weeks of personal vacation per year	
<1	2%
1-2	7%
3-4	49%
5-6	17%
7-8	22%
>9	3%
Days away from practice for continuing education	
0	2%
<3	0%
3-7	24%
8-14	51%
15-21	15%
22-28	5%
>28	3%

### Scope of practice and resources

At the time of the survey, only 17% of surgeons reported practicing exclusively rhinology, dedicating 100% of their operative time to rhinology. An additional 31% focused at least 75% of available time and resources on rhinologic cases. Most surgeons (81%) operate 1 or 2 days a week. Overall, 42% felt their operating room (OR) resources were insufficient to meet their patient care need. No surgeons described an excess of operative resources.

When evaluating endonasal surgical approaches, 63% reported using endoscopic approaches in greater than 80% of cases. Ninety four percent (94%) of surgeons had access to image guidance systems (IGS). Fifty eight percent (58%) of surgeons reported performing in office procedures, with 14% performing over 60 in-office cases per year.

In an effort to quantify the case load of a rhinologic surgeon, surgeons were asked to quantify the number of new rhinology consults and operative cases. Surgeons reported an average of 58 new inpatient consults per year, with an average of 818 in office consults per year. The average number of new operative cases is described in Table 5. Respondents reported an average of 73 primary endoscopic sinus surgeries (ESS) per year and an average of 59 revision ESS cases per year.

**Table 5:** **Scope of Practice**

OR days per week	
<1/week	17%
1/week	42%
2/week	39%
3/week	2%
% of OR time dedicated to rhinology	
<25%	2%
26%-50%	25%
51%-75%	25%
75%-99%	31%
100%	17%
Current OR time	
Too much	0%
Appropriate	58%
Too little	42%
% of cases performed endoscopically	
1-20%	9%
21-40%	6%
41-60%	11%
61-80%	11%
81-100%	63%
% of cases performed with IGS	
0%	11%
1-20%	20%
21-40%	17%
4 1-60%	11%
61-80%	9%
81-100%	26%
No IGS available	6%
Number of in office procedures per year	
None	42%
1 to 20	20%
20 to 40	16%
40 to 60	8%
Greater than 60	14%
Number of new rhinology consults per year (SD)	
Inpatient/ER	58 (22.3)
In office	818 (152.3)
Number of operative procedures per year (SD)	
Primary Endoscopic Sinus Surgery (CRS)	73 (24.1)
Revision Endoscopic Sinus Surgery (CRS)	59 (18.1)
Endoscopic resection of benign sinonasal neoplasms	14 (3.9)
Septoplasty	125 (40.4)
Endoscopic pituitary adenoma resection	14 (5.1)
Anterior Skull Base resection (benign)	4 (1.5)
Anterior Skull base resection (malignant)	3 (1.3)
Orbital Decompression (Grave’s)	2 (1.0)

*CRS* - Chronic Rhinosinusitis.

*SD* - Standard Deviation.

*IGS* - Image Guidance System.

### Job satisfaction, income, and future practice plans

Overall physician wellbeing and satisfaction is being increasingly emphasized as poor physician health has been suggested to adversely affect health care systems [13,14]. The majority of surgeons surveyed were satisfied with their careers (83%). Sixty six percent (66%)of surgeons reported being satisfied with their current income. The reported incomes and overhead costs are reported in Table 6.

**Table 6:** **Job Satisfaction, Income, Future Practice Plans**

Satisfaction	
Very dissatisfied	8%
Somewhat dissatisfied	6%
Neutral	3%
Somewhat satisfied	33%
Very satisfied	50%
Income	
< $200,000	8%
$200,000 to $400,000	24%
$400,001 to $600,000	37%
$600,001 to $800,000	16%
> $800,000	5%
Undisclosed	10%
Income Satisfaction	
Satisfied	66%
Neutral	26%
Dissatisfied	8%
Relocation	
Definitely not moving	46%
Unlikely to move	37%
Not sure	14%
Probably moving	3%
Definitely moving	0%
Average Planned retirement age (SD)	66 (4.0)

*SD* – Standard Deviation.

Eighty three percent (83%) of respondents reported no plan to relocate their practice. The average planned retirement age was 66 (SD = 4.0) years of age, which was 20 years from the average age of surveyed physicians. Eighty six percent (86%) of physicians planned to retire at an age greater than 60.

### Waiting times and workforce assessment

Surgeons were asked to state the maximal acceptable waiting time and percentage of patients who receive surgery by the maximum acceptable wait time. Results are described in Table 7. Surgeons reported only 56% (range =44% to 63%) of patients receiving surgery within the maximum acceptable wait time, regardless of the procedure.

**Table 7:** **Waiting Times Assessment**

	**Maximal acceptable waiting time (weeks) (SD)**	**Percent of patients who receive surgery within maximal acceptable waiting times (SD)**
Primary Endoscopic Sinus Surgery (CRS)	15 (8.5)	45% (38.9)
Revision Endoscopic Sinus Surgery (CRS)	16 (14.2)	44% (36.7)
Endoscopic resection of benign sinonasal neoplasms	11 (9.7)	63% (33.9)
Septoplasty	30 (31.7)	53% (37.7)
Endoscopic pituitary adenoma resection	10 (10.1)	55% (35.4)
Anterior Skull Base resection (benign)	10 (9.9)	60% (37.8)
Anterior Skull base resection (malignant)	4 (5.1)	66% (39.9)
Orbital Decompression (Grave’s)	8 (10.4)	60% (34.4)

*CRS* - Chronic Rhinosinusitis.

*SD* - Standard Deviation.

Table 8 summarizes the respondents’ opinions regarding the current rhinologic workforce and factors influencing the delivery of care. When asked what factors affected delivery of timely care, 54% of surgeons reported that insufficient OR time was often or always a factor. 63% felt that prolonged surgical waitlists was a significant factor in reducing the quality of care to patients.

**Table 8:** **Workforce Assessment**

Factors affecting delivery of care*	
Prolonged clinic wait times	57%
Delay in investigations	16%
Prolonged surgical waitlists	63%
Insufficient OR time	54%
# of Surgeons practicing rhinology	
Too Few	18%
Appropriate	68%
Too Many	14%
# of fellowship-trained Rhinologists	
Too Few	23%
Appropriate	71%
Too Many	6%
Training too many Rhinologists in North America	
Yes	42%
No	19%
Unsure	39%
Too many Rhinology fellowships in Canada?	
Yes	19%
No	53%
Unsure	28%
Advised residents not to pursue rhinology due to perceived lack of jobs	
Yes	17%
No	83%
Appropriate ratio of Rhinologists to population (median, mode)	1:500,000
Average additional required Rhinologists per region	1
Institutions currently seeking Rhinologist	6%
Institutions planning on recruiting in the next 2-5 years	31%

*often or always.

Currently, 68% and 71% of the respondents feel the number of surgeons practicing rhinology and the number of fellowship-trained rhinologists is appropriate, respectively. However, 23% feel there is a shortage of fellowship-trained rhinologists. Only 6% felt there were too many fellowship-trained rhinologists. Regarding training patterns, 42% felt too many rhinologists were being trained in North America. In contrast, only 19% felt too many rhinologists were being trained in Canada. Seventeen percent (17%) of surgeons reported advising residents not to pursue rhinology due to perceived lack of jobs. Overall, there appears to be diverse opinions regarding the current rhinologic workforce and the perceived need for future rhinologists in Canada.

Respondents suggested an appropriate rhinologist to population ratio would be 1:500,000. Currently, only 2 respondents reported that their institution was seeking an additional rhinologist. Thirty one percent (31%) believe their institution will recruit an additional rhinologist in the next 2-5 years.

## Discussion

The objective of this study was to define the demographics and current practice patterns of the Canadian Rhinologic Workforce. Of the otolaryngologists identified as having a practice comprised of at least 50% rhinology, 65% responded to the invitation to participate in the survey. Generally, a 40% response rate represents an average survey response rate – greater than 60% response is considered excellent in email and online surveys [15-17]. Given the excellent response rate, the outcomes from this survey are likely representative of the Canadian rhinologist population.

Supplying society with the appropriate health care in a timely and accessible manner requires accurate matching of societal needs with labor capacity. Therefore, workforce planning is a challenging but important component to improving the overall quality of health care delivery in Canada. Although several contributing factors to the current surgical labor inefficiencies have been proposed, such as increasing female surgeon workforce or generational differences in lifestyle demands, the first step toward improving workforce planning is to define the current labor characteristics [8,12,18,19]. The results of this survey demonstrate that the majority of rhinologists are male with an average age of 46. This reflects a persistent male prevalence in the surgical workforce [12,20,21]. There is a perception that female surgeons will be less productive than their male counterparts, thus contributing to a potential workforce shortage [8,22]. There is little doubt that the younger generations of surgeons are placing more emphasis on lifestyle than previous generations, however, the authors agree with Pillsbury who states: “any difference in proposed productivity based on gender is inappropriate” [18]. There is no current evidence to support this opinion, and previous literature that suggested a difference is outdated [19]. If there is a significant difference in the output of men and women, it more likely reflects society norms, and is unlikely to significantly affect workforce projections [19,23].

The age of the respondents is similar to that reported in the thoracic surgery workforce assessment, which echoes a somewhat younger surgeon workforce population [12]. The planned average age of retirement is 66. While this represents an older age of retirement than the general population, it is similar to that of US surgeons [10]. This delay in retirement may be linked to variations in the economic market over recent years and certainly affects workforce planning. As surgeons continue to work longer, the creation of new opportunities is delayed and this contributes to the perceived and actual lack of employment.

The majority of respondents completed a fellowship in rhinology (83%). The average year of beginning practice for non-fellowship trained rhinologists was 1991 while the average year of fellowship-trained rhinologists was 2005. This is in line with an increasing trend for residents to pursue additional training after residency and may be related to a number of factors. First, the Royal College of Physicians and Surgeons employment survey suggests that this may be due to trainees’ perceptions that additional training will make them more employable. Second, it is also possible that residents are pursing fellowships as an alternative to unemployment [2]. Lastly, it may be related to the fact that rhinology is a relatively young subspecialty with opportunities significantly increasing over the last 20 years. A recent survey study of Canadian otolaryngology residents had similar findings - 78% of respondents planned to pursue a fellowship and 90% stated this decision was moderately influenced by limited job options. Only 22% of graduating residents had confirmed employment. This study was performed several months prior to graduation and as some residents may have found positions closer to graduation, this may overestimate the degree of unemployment of Canadian otolaryngology residents [11].

Rhinologists appear to have reasonable work hour requirements compared to other surgical specialties. Only 25% of respondents work more than 60 hours per week, compared to 82% of thoracic surgeons [12]. Only 2% of surgeons reported a call frequency of more than 1 in 4. The current division of work responsibilities seems to be reasonably balanced, creating optimal conditions to prevent physician burnout [24]. Along these lines, rates of physician satisfaction both with careers and remuneration were high (>80%). These high rates of physician satisfaction are higher than those found in general surgery, orthopedic surgery and even ophthalmology [25].

Canada’s relatively long waitlists for medical care is a well known issue [4]. More specifically, wait times for elective endoscopic sinus surgery for refractory chronic rhinosinusitis are among the longest with patients waiting between 6 to 12 months for surgery [26]. Prolonged waitlists are associated with tremendous costs, as well as adverse patient effects [6,7,27-29]. The survey respondents echoed these concerns. Surgical resources were thought to be a major limiting factor in providing timely care. Forty two percent felt their current OR time was inadequate and 63% suggested prolonged surgical waitlists often delayed care. Surgeons reported only 56% of patients received care within their maximal acceptable waiting time for common rhinologic procedures. Of note, the second most common reason for untimely care was prolonged clinical wait lists. This is consistent with reports of increasing referral wait times and emphasizes that while increasing operative time would shorten surgical waitlists, it may not improve Canadian’s access to specialist consultations [4,7]. An increasing number of surgeons are performing in office procedures (58%), which may be a direct result of these waitlists. What procedures may be performed safely, their outcomes, and how this will affect surgical waitlists, are areas of ongoing study.

Assessments of the US otolaryngology workforce suggest an impending workforce crisis, projecting a deficit of over 2,000 otolaryngologists by 2025 [8,9]. Although we cannot accurately extrapolate and apply US-based workforce data toward Canadian workforce projections, why is there a perceived ‘job crisis’ for Canadian otolaryngologists given the well-documented issue of prolonged patient waitlists? This, unfortunately, would seem to be a result of a mismatch between the workforce needs and the Canadian health care system capacity/prioritization of resources. Increasing capacity is a complex and costly problem that is the focus of health services research but it undeniably needs to be addressed, and hopefully soon. The deficits in the accessibility and timeliness of care result in a significant decrease in the quality of care patients receive [5]. A formal workforce assessment, which is the next step in this analysis, will help define societal needs and provide a guide for capacity planning and resource allocation.

There are several limitations of this study that must be considered when interpreting the results. First, this study involved the use of a non-validated survey. Currently, there is no validated survey available for assessing surgical demographics for the use in future workforce planning. However, this survey has been used previously for surgical workforce planning and is therefore consistent with other studies in this area. Additionally, the modification via the Delphi method strengthens the rhinology related questions ensuring important topics specific to rhinologists are discussed. Second, data collection was performed using an electronic platform with online responses. While a 65% response rate is considered excellent for any electronically administered survey, the respondents are potentially biased to a more technologically inclined generation and therefore a potentially younger cohort. Third, while the results of the study may be valid for the country as a whole, they may not be generalizable to specific high or low demand regions. Fourth, several of the questions pertaining to surgical waitlist and regional need for another rhinologist are opinion-based rather then being objectively measured, which may result in a reporting bias. Finally, this study was limited to surgeons practicing >50% rhinology and thus does not capture all rhinologic practices in Canada, for example general otolaryngologists performing rhinologic procedures. However, surveying this population captures the demographics and practice patterns of surgeons that will have the most influence on workforce needs. Despite these limitations, this is the most robust attempt to define to Canadian Rhinologic workforce and provides valuable data for future workforce modeling.

## Conclusion

This nationwide survey study defined the demographics, education, practice patterns, and surgeons attitudes toward workforce requirements and training of the current Canadian Rhinologic workforce. The rhinology workforce does not appear to be saturated and, combined with the current state of prolonged waitlists and poor timeliness of care, suggests that there is a potential need for more rhinology subspecialists in certain regions of Canada. This data will facilitate future rhinologic workforce planning to define population needs and identify possible solutions to these deficits.

## Abbreviations

CSO: Canadian Society of Otolaryngology-Head and Neck Surgery
OR: Operating room
IGS: Image guidance systems
ESS: Endoscopic sinus surgery
SD: Standard deviation
USA/US: United States of America
ARS: American Rhinologic Society
ERS: European Rhinologic Society
CRS: Chronic Rhinosinusitis.
